# A Few Thoughts on the Combination of Filling-Materials

**Published:** 1893-08

**Authors:** Benjamin Lord

**Affiliations:** New York


### A FEW THOUGHTS ON THE COMBINATION OF FILLING-MATERIALS.[1]

BY DR. BENJAMIN LORD, NEW YORK.

Mr. President and Members,—A great deal has been said and written, from time to time, on the subject of combining in some way different filling-materials.

The idea, or the practice, is not exactly new, but more has been said about it of late than formerly; and, as all are aware, the sub-

[1] Read before the New York Odontological Society, May 16, 1893.

ject has been discussed not a little before this Society in the last few years.

There has been less said about the combination of foils than about the combining of amalgam and gold, and the use of oxyphosphate with gold, and also with amalgam. For some reason which I do not claim to understand, we find that when amalgam is put by the side of gold, in cases where decay has extended beyond the gold filling, it does better; that it changes less in the way of shrinkage, and that the edges or margins are maintained much more perfectly than when amalgam only is used.

I have not tried the packing of gold and amalgam together, and so cannot speak from experience about it; but I should apprehend a good deal of embarrassment and risk in the attempt to pack gold by the side of, or into, a soft material. I have felt that in cavities in which, for any reason, I wished to use gold and amalgam in combination, a much better practice would be to make the gold filling first,—not being particular to pack the gold solid at that point or part of the cavity where I propose to put amalgam,—and then, after condensing and finishing the surface and margins, to remove the gold from that part of the cavity intended for the amalgam, and to pack the latter by the side of the gold. The effect of this would be the same as the packing of amalgam by the side of gold to arrest further decay, as before alluded to. I believe that such a method of combining the two materials will be found highly satisfactory.

The combining of alloy filings with oxyphosphate I find in my practice to be very useful. The filling lasts longer and the color is better.

My belief is, I may say, that the use of oxyphosphate in the starting or retaining of gold fillings, as recommended by some, could not be a certain or reliable practice, for very good and logical reasons.

A combination in which I find great interest is in the use of soft or non-cohesive gold with tin-foil. This is no novelty in practice; but I think that, for the most part, too great a proportion of tin has been used, and hence has arisen the objection that the tin dissolved in some mouths. I am satisfied that I myself, until recently, employed more tin than was well. I now use from one-tenth to one-twelfth as much tin as gold, and no disintegration or dissolving away of the tin ever occurs. I fold the two metals together, in the usual way of folding gold to form strips, the tin being placed inside the gold. The addition of the tin makes the gold tougher, so that it works more like tin-foil. The packing can be done with more ease and certainty; the filling, with the same effort, will be harder; and the edges or margins are stronger and more perfect.

The two metals should be thoroughly incorporated by manipulation. Then, after a time, there will be more or less of an amalgamation. By using about a sixteenth of tin, the color of the gold is so neutralized that the filling is far less conspicuous than when it is all gold; and I very often use such a proportion of tin in cavities on the labial surfaces of the front teeth.

If too much tin is employed in such cases, there will be some discoloration of the surface of the fillings; but in the proportion that I have named, no discoloration occurs, and the surface of the filling will be an improvement on gold in color.

There is another combination in which I find great interest and advantage. It is the using of non-cohesive and cohesive gold, by folding the two together in the proportion of one-third cohesive to two-thirds non-cohesive. I first fold the non-cohesive once, then lay it on the strip of cohesive gold, and fold the two together; the folding thus secures the cohesive always on the outside.

Gold prepared in this way should be used as soft gold, and works almost exactly like soft or non-cohesive gold in those qualities in which soft gold is superior to cohesive; but it is tougher, packs more readily, makes a more solid filling, and gives stronger and better margins.

